# Degradation of Alginate by a Newly Isolated Marine Bacterium *Agarivorans* sp. B2Z047

**DOI:** 10.3390/md20040254

**Published:** 2022-04-04

**Authors:** Xun-Ke Sun, Ya Gong, Dan-Dan Shang, Bang-Tao Liu, Zong-Jun Du, Guan-Jun Chen

**Affiliations:** 1 Marine College, Shandong University, Weihai 264209, China; sunxunke1994@163.com (X.-K.S.); 201916548@mail.sdu.edu.cn (D.-D.S.); 15069542048@163.com (B.-T.L.); duzongjun@sdu.edu.cn (Z.-J.D.); 2 State Key Laboratory of Microbial Technology, Shandong University, Qingdao 266237, China

**Keywords:** alginate-degrading strain, alginate lyase, *Agarivorans* sp. B2Z047, bioconversion, marine bacterium

## Abstract

Alginate is the main component of brown algae, which is an important primary production in marine ecosystems and represents a huge marine biomass. The efficient utilization of alginate depends on alginate lyases to catalyze the degradation, and remains to be further explored. In this study, 354 strains were isolated from the gut of adult abalones, which mainly feed on brown algae. Among them, 100 alginate-degrading strains were gained and the majority belonged to the *Gammaproteobacteria*, followed by the *Bacteroidetes* and *Alphaproteobacteria*. A marine bacterium, *Agarivorans* sp. B2Z047, had the strongest degradation ability of alginate with the largest degradation circle and the highest enzyme activity. The optimal alginate lyase production medium of strain B2Z047 was determined as 1.1% sodium alginate, 0.3% yeast extract, 1% NaCl, and 0.1% MgSO_4_ in artificial seawater (pH 7.0). Cells of strain B2Z047 were Gram-stain-negative, aerobic, motile by flagella, short rod-shaped, and approximately 0.7–0.9 µm width and 1.2–1.9 µm length. The optimal growth conditions were determined to be at 30 °C, pH 7.0–8.0, and in 3% (*w*/*v*) NaCl. A total of 12 potential alginate lyase genes were identified through whole genome sequencing and prediction, which belonged to polysaccharide lyase family 6, 7, 17, and 38 (PL6, PL7, PL17, and PL38, respectively). Furthermore, the degradation products of nine alginate lyases were detected, among which Aly38A was the first alginate lyase belonging to the PL38 family that has been found to degrade alginate. The combination of alginate lyases functioning in the alginate-degrading process was further demonstrated by the growth curve and alginate lyase production of strain B2Z047 cultivated with or without sodium alginate, as well as the content changes of total sugar and reducing sugar and the transcript levels of alginate lyase genes. A simplified model was proposed to explain the alginate utilization process of *Agarivorans* sp. B2Z047.

## 1. Introduction

As an important sustainable renewable resource, brown algae provide a huge biomass across the global ocean, from kelp forests to algae blooms to seaweeds cultivation [1]. The main component of the cell wall of brown algae is alginate, a kind of water-soluble acidic polysaccharide composed of β-d-mannuronate (M) and α-l-guluronate (G), generating three block types: homopolymeric M-residues (polyM), homopolymeric G-residues (polyG), and randomly alternating M- and G-residues (polyMG) [2,3]. The prerequisite of efficient bioconversion of brown algae is the degradation of alginate, catalyzing by alginate lyases and producing alginate oligosaccharides. Alginate oligosaccharides can be used as food for aquatic animals, elicitors of plants and microorganisms, prebiotics, and cryoprotectors [4]. Thus, alginate lyases play key roles in the full utilization of brown algae and the effective production of alginate oligosaccharides, and have become a hot topic.

Alginate lyases have been isolated from marine organisms, such as algae, marine molluscs, marine and terrestrial bacteria, fungi, and viruses [5]. Among them, marine microorganisms are the primary source of alginate lyases, and alginate lyase genes have been found in strains belonging to the genera *Sphingomonas*, *Vibrio*, *Pseudomonas*, *Microbulbifer*, *Pseudoalteromonas*, *Formosa*, *Flavobacterium*, *Agarivorans*, *Flammeovirga*, *Shewanella*, *Marinimicrobium*, *Zobellia*, and *Agrobacterium* [6]. Most of the elucidated enzymes show endolytic activities, and only a few have been reported as exolytic lyases. The specific activities and degradation rate of most currently reported exolytic alginate lyases were relatively low owing to its single degradation pattern and substrate preferences [7,8,9,10]. Based on the substrate specificity, alginate lyases are classified into M-specific lyase, G-specific lyase, bifunctional MG lyase, and alginate oligosaccharide lyase [11]. According to the polysaccharide lyase family category in the CAZy database (www.cazy.org, accessed on 26 March 2022), alginate lyases are discovered in many PL families, such as PL5, PL6, PL7, PL14, PL15, PL17, PL18, and the recently identified families PL31, PL32, PL34, PL36, and PL39. For example, PL6 and PL7 family members possess different substrate specificities and are able to degrade all types of alginates, while PL17 family proteins mainly belong to alginate oligosaccharide lyase [12,13,14]. To date, only two PL38 proteins, CUL-I and TpPL38A, have been characterized as endo-β-1,4-glucuronan lyase, and CUL-I does not act on alginate [15,16]. In the CAZy database, 1339 proteins are categorized as PL38 family members, whereas some proteins have been annotated as alginate lyases by the NCBI Prokaryotic Genome Annotation Pipeline, such as an alginate lyase family protein (QIH42118.1) from *Vibrio ziniensis*. Meanwhile, an alginate lyase (ALJ44842.1) from *Bacteroides ovatus* has been structured, and the related article is to be published. Thus, PL38 family proteins may possess the alginate lyase activity, while this enzyme activity has not been proven by experiments until now. In addition, it is feasible to directly use microbial fermentation to produce alginate oligosaccharides [17,18,19,20]. Therefore, it is still an important direction to screen alginate-degrading bacteria and alginate lyases suitable for industrial production.

Marine mollusks, feeding on algae, have been widely identified by researchers as an important source for screening alginate-degrading bacteria. The guts of marine gastropods have been found to be enriched in alginate-degrading bacteria [21]. Microbes in the gut of abalones could help their hosts promote the nutrient absorption by degrading alginate [22]. The abalone mainly feed on macroalgae, such as brown algae and red algae, and adult *Haliotis discus hannai* mainly feed on brown algae [23]. Tanaka’s study showed that 70% of all strains isolated from the abalone gut could degrade alginate [24]. The genus *Agarivorans* was first established in 2004 [25], and four species isolated from marine mollusks, seawater, and tidal flat have been published. Since the establishment of this genus, a large amount of research has focused on the agarase produced by *Agarivorans* strains [26,27,28]. Here, 354 strains were isolated from the gut of adult abalones and screened for the degradation of alginate. The alginate-degrading capability was determined by the transparent circle diameter around the colonies and the enzyme activity of alginate lyase in the cell free supernatant. Meanwhile, the alginate-degrading strains were classified to a genus level based on the 16S rRNA gene sequencing. The highly efficient alginate-degrading bacterium, *Agarivorans* sp. B2Z047, was gained and the production conditions of the alginate lyase by strain B2Z047 were investigated. Bioinformatics analyses were performed for all 12 alginate lyases from the genome of strain B2Z047, and they were overexpressed in *E. coli* to characterize the enzyme activity. Furthermore, the key alginate lyases functioning in the degradation of alginate were determined by the real-time fluorescence quantitative PCR.

## 2. Results and Discussion

### 2.1. Isolation and Cultivation of Alginate-Degrading Strains

**Figure 1:**
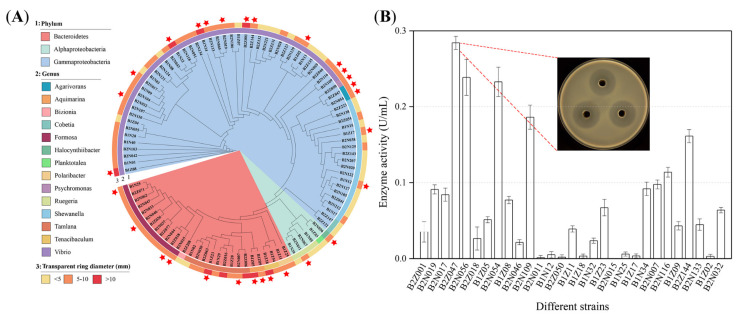
Screening of the alginate-degrading bacteria. (**A**) Maximum-likelihood phylogenetic tree based on the 16S rRNA gene sequences of 100 alginate-degrading strains. From the inside to the outside, 1, the classification of the phylum; 2, the classification of the genus; 3, the diameter of degradation circle. The strains labeled with red star were selected for further analysis. (**B**) The enzyme activity of 29 selected strains. Strains with a larger degradation circle were cultivated in MB containing alginate as the sole carbon source for 48 h, and then enzyme activity of alginate lyase in the cell free supernatant of each strain was determined by the DNS method. The degradation circle of strain B2Z047 was displayed, which showed that its alginate lyases might degrade polyM (white-halo) and polyG (white-ring) [34].

To obtain the highly efficient alginate-degrading strains, the gut of adult abalones was sampled using a standard dilution plating technique on marine agar 2216 (MA). A total of 354 strains were gained and screened for the degradation of alginate on the modified MA medium containing 0.5% sodium alginate. Among these 354 strains, 100 strains possessed the transparent ring around their colonies, resulting from the degradation of alginate in the solid plates (Figure 1A, B). Nine strains held the large transparent ring with the diameter > 10 mm, 60 strains harbored the transparent ring of the diameter between 5 mm and 10 mm, and the remaining strains presented the small transparent ring (<5 mm), meaning that these strains might degrade alginate.

Meanwhile, the 16S rRNA gene sequences of these 100 strains were sequenced to recognize the selected alginate-degrading bacteria (Data S1, Supplementary Materials). The sequence alignments showed that the majority of the alginate-degrading strains belonged to the *Gammaproteobacteria*, followed by the *Bacteroidetes* and *Alphaproteobacteria* (Figure 1A). At the genus level, the alginate-degrading strains of genus *Vibrio* were the most prominent, accounting for 44% of the total bacteria. Other dominant strains belonged to the genera of *Shewanella* (17%), *Formosa* (12%), *Tamlana* (9%), and *Ruegeria* (4%). Several genera, *Agarivorans*, *Aquimarina*, *Cobetia*, *Polaribacter*, and *Tenacibaculum*, were obtained as a percentage of 2%. The other four strains could be classified into the genera *Bizionia*, *Halocynthiibacter*, *Planktotalea*, and *Psychromonas*, respectively. Additionally, 30 strains might be the potential novel species due to the highest 16S rRNA similarity below the species threshold value of 98.7%.

A total of 29 strains with the potential to degrade alginate efficiently were selected to further examine the degradation ability (labeled with red star in Figure 1A). These strains were cultivated in the marine broth 2216 (MB) for 48 h, and then the cell free supernatant of each strain was used to determine the enzyme activity of alginate lyase by the 3,5-dinitrosalicylic acid (DNS) method [29]. The results showed that strain B2Z047 had the strongest capacity of degrading alginate (0.28 ± 0.01 U/mL), followed by the strains B2N056 and B2N054 (Figure 1B). Both the B2Z047 and B2N054 strains belonged to the genus of *Agarivorans*, and the documents involved in the degradation of alginate by *Agarivorans* strains were very few [30,31,32,33]. Therefore, the alginate lyase production and the potential alginate-degrading mechanism of *Agarivorans* sp. B2Z047 were investigated.

### 2.2. Optimization of the Production Conditions of the Alginate Lyase by Agarivorans sp. B2Z047

**Figure 2:**
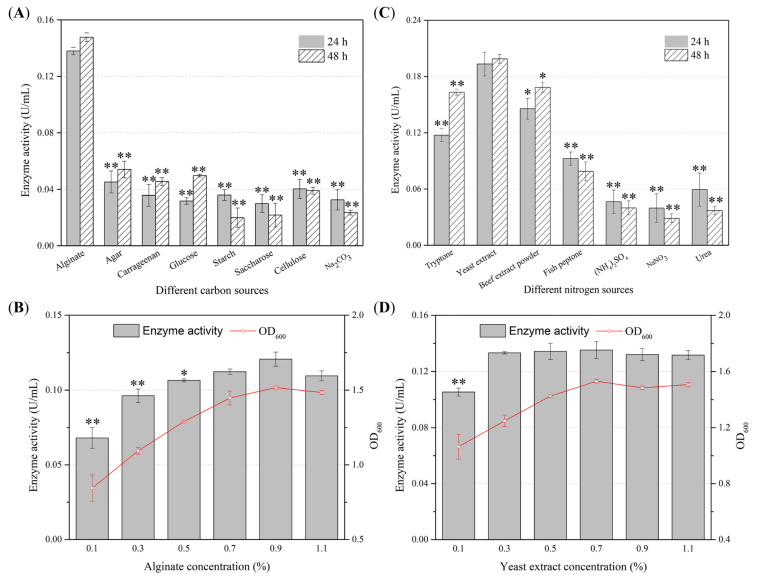
Cultured medium optimization for the alginate lyase production by *Agarivorans* sp. B2Z047. (**A**) Effects of different carbon sources on the production of alginate lyase. Each carbon source (0.5%, *w*/*v*) was added into the medium containing 0.5% peptone in artificial seawater (pH 7.0), and alginate lyase activity in the cell free supernatant was measured after 24 and 48 h cultivation. (**B**) Alginate lyase activity and cell density (optical density at 600 nm [OD_600_]) in different concentrations of sodium alginate supplemented medium were determined after 24 h culture. (**C**) Alginate lyase activity in the supernatant of 24 and 48 h cultures of different nitrogen sources added. The medium containing 0.5% sodium alginate in artificial seawater (pH 7.0) was individually supplemented with tryptone (0.5%), yeast extract (0.5%), beef extract powder (0.5%), fish peptone (0.5%), (NH_4_)_2_SO_4_ (0.28%), NaNO_3_ (0.36%), or urea (0.06%). (**D**) Alginate lyase activity and cell density in different concentrations of yeast extract added media were detected after 24 h cultivation. Three replications were performed for each experiment. The error bars represent the standard deviation, and asterisks denote *p*-values for *t*-tests of differences from the data of alginate (**A**), yeast extract (**B**), 0.9% alginate (**C**), and 0.3% yeast extract (**D**): * *p* < 0.05, ** *p* < 0.01.

To obtain the optimal alginate lyase production condition, the effects of carbon sources, nitrogen sources, and metal ions on enzyme production of strain B2Z047 were investigated by single factor experiment. When sodium alginate was used as the sole carbon source, the enzyme activities of alginate lyase in the cell free supernatant after 24 and 48 h cultivation were the highest, while the presence of other carbon sources could not be helpful (Figure 2A). After determining sodium alginate as the optimal carbon source, effects of different concentrations of sodium alginate on the alginate lyase production were explored (Figure 2B, measured at 24 h). It was found that when the concentration of sodium alginate in the medium was 0.9%, the alginate lyase activity was the highest. However, the enzyme activities between the 0.9% and 0.7% (*p* = 0.08), or 0.9% and 1.1% (*p* = 0.05) sodium alginate supplemented medium were not significantly different. The viscosity of the medium might increase with more sodium alginate added, resulting in the lack of dissolved oxygen in the broth, which was probably bad for the growth and reproduction of bacteria. Thus, in the 0.9% sodium alginate medium, the cell density of strain B2Z047 also reached a peak, with a positive correlation between enzyme activity and bacterial biomass. Nitrogen is an indispensable element for the growth of microorganisms. We further explored the influences of four organic nitrogen sources and three inorganic nitrogen sources on alginate lyase production of *Agarivorans* sp. B2Z047 (Figure 2C). When yeast extract was added as a nitrogen source, the alginate lyase activity in the supernatant was the highest, and the enzyme activities were higher, with more than 0.3% yeast extract supplemented (Figure 2D).

The addition of MgSO_4_ in the medium was found to enhance the alginate lyase production of strain B2Z047, while the presence of ZnSO_4_, NiSO_4_, FeSO_4_, and CuSO_4_ lowed the alginate degradation activity (Figure S1A, Supplementary Materials). When compared with the control, the supplement of CaCl_2_ (*p* = 0.49 after 24 h cultivation) or KH_2_PO_4_ (*p* = 0.36 after 24 h cultivation) did not alter the enzyme yield. The enzyme activity in the EDTA added medium was significantly lower than that in other medium, meaning that ions were essential to the production or the function of alginate lyases. The impacts of different concentrations of MgSO_4_ on the degradation activity of alginate of strain B2Z047 were determined, and the results showed that the enzyme activity of alginate lyase was higher with 0.05%, 0.075%, or 0.1% MgSO_4_ supplemented (Figure S1B). In addition, when NaCl was added into the medium, alginate lyase production was increased (Figure S1C). The enzyme production differences among the initial pH from 6 to 9 of the culture media were not observed (Figure S1D). Subsequently, the influences of other factors, i.e., culture temperature, rotating speed of shaker, bottling volume in the flask, and seed volume, on the enzyme production were investigated (Figure S1E–H). The results showed that the optimal bottling volume was 50 mL in the 250 mL flask; the culture temperatures 25, 28, and 33 °C were better than others; the rotation rates 125, 150, 175, and 200 rpm were better than 225 rpm; and the inoculum 0.5, 1, and 2 mL were more effective. Furthermore, the orthogonal experiment was designed to eliminate the interaction among different components. The effects of four factors and three levels on enzyme production were investigated. The results showed that the optimal alginate lyase production medium was 1.1% sodium alginate, 0.3% yeast extract, 1% NaCl, and 0.1% MgSO_4_ in artificial seawater (pH 7.0, Table S1), and strain B2Z047 showed the enzyme activity of 0.50 ± 0.04 U/mL after 24 h cultivation.

### 2.3. Characterization of Strain B2Z047

**Figure 3:**
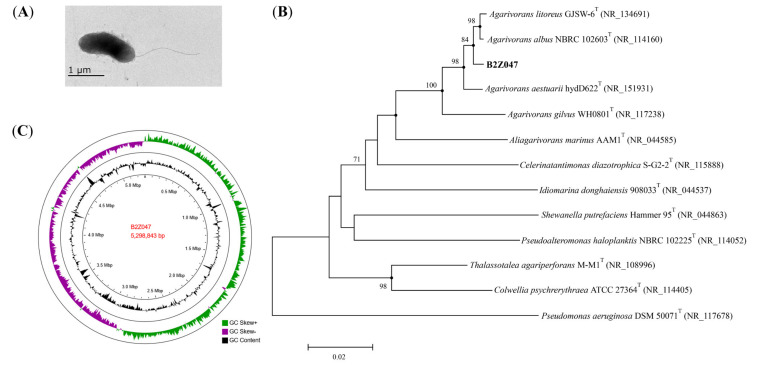
Characterization of *Agarivorans* sp. B2Z047. (**A**) Transmission electron microscopy of cell of strain B2Z047. Cells were grown on MA at 30 °C for 48 h. Bar, 1 µm. (**B**) Neighbor-joining phylogenetic tree based on the 16S rRNA gene sequences, showing the relationships of strain B2Z047 (GenBank: OM278383) and the related species. Bootstrap values ≥ 70% (based on 1000 replications) were shown at branching nodes. Filled circles indicated that the corresponding nodes were also recovered in the trees generated with the maximum-likelihood and maximum-parsimony algorithms. *Pseudomonas aeruginosa* DSM 50071^T^ was used as an out-group. Bar, 0.02 substitutions per nucleotide position. (**C**) Genome map of strain B2Z047. The circles from the inner to the outer: Circle 1 for GC content; and Circle 2 for GC skew.

Cells of strain B2Z047 were Gram-stain-negative, aerobic, motile by flagella, short rod-shaped, and approximately 0.7–0.9 µm width and 1.2–1.9 µm length (Figure 3A). Colonies on MA plate were circular, entire convex, smooth, yellowish white, and approximately 3 mm in diameter after incubation for two days at 30 °C (Figure S2). Growth occurred at 4–37 °C (optimum, 30 °C), at pH 6.0–10.0 (optimum, 7.0–8.0), and in the presence of 1–8% (*w*/*v*) NaCl (optimum, 3%). Nitrate was reduced to nitrite. Strain B2Z047 had various carbohydrate degradation enzyme activities, such as amylase, agarase, alginate lyase, and cellulase activity. Catalase, oxidase, and gelatinase were positive, but no esterase activity was detected.

Strain B2Z047 was found to be most close to *Agarivorans litoreus* with the 16S rRNA gene sequence similarity of 98.8%, followed by *A. aestuarii* (98.7%), *A. albus* (98.4%), and *A. gilvus* (96.3%). The phylogenetic tree was constructed by the 16S rRNA gene sequences, and strain B2Z047 clustered in the clade of the genus *Agarivorans* (Figure 3B). The draft genome of strain B2Z047 (WHRY00000000) was assembled with the total sequence length of 5,243,710 bp and containing 57 scaffolds, coverage of 100.0×, scaffold N50 value of 466,185 bp. By the NCBI Prokaryotic Genome Annotation Pipeline (PGAP), 4824 genes were annotated. Among them, 4642 protein-coding genes and 99 RNA genes were identified. Furthermore, the complete genome sequencing of B2Z047 yielded a genome of 5,298,843 bp in length after assembly, and sequencing depth of coverage was 124.8× (CP088080, Figure 3C). The DNA G+C content of B2Z047 calculated from the complete genome sequence was 44.1%, which was similar with the genomes of *Agarivorans* genus. The complete genome of B2Z047 contained 5014 genes, 4853 protein-coding genes, 22 rRNAs, and 93 tRNAs.

The phylogenomic analyses were performed, and the average nucleotide identity (ANI) and the digital DNA–DNA hybridization (dDDH) values were calculated. The ANI values between genomes of strain B2Z047 and *A. litoreus*, *A. aestuarii*, and *A. albus*, three strains with the 16S rRNA gene sequence similarity more than 98%, were 93.0%, 91.7%, and 97.1%, respectively. The dDDH values were 49.7%, 44.6%, and 75.0%, respectively. The genomes of strain B2Z047 and *A. litoreus*, most similarity of the 16S rRNA gene sequences, exhibited lower ANI and dDDH values, which were less than the threshold of 95–96% (ANI) and 70% (dDDH) for species delineation. However, the genomes of strain B2Z047 and *A. albus* showed higher ANI and dDDH values, which exceeded the threshold for species delineation. These results indicated that strain B2Z047 is close to *A. litoreus* with the 16S rRNA gene sequence similarity of 98.8%, but similar to *A. albus* with the high ANI and dDDH values. Thus, the systematic status of strain B2Z047 need further, more detailed studies.

### 2.4. Sequence Analyses of Alginate Lyases

Based on the whole genome sequencing of strain B2Z047, a total of 12 alginate lyase genes, 87 genes encoding carbohydrate binding modules, 194 glycosyltransferase genes, 180 glycoside hydrolase genes, and 30 carbohydrate esterase genes have been annotated by CAZy database. These 12 potential alginate lyase genes were scattered in the genome of strain B2Z047 (Table 1). Among these genes, only *oal17A1* (*2231*) and *oal17A2* (*2232*) were close together, while *aly7A2* (*2288*) and *aly7A3* (*2290*) were separated by one gene encoding the phosphodiesterase. These neighboring genes might be functionally related and controlled coordinately. According to the annotation, Aly6A belonged to PL6 family, Aly38A was affiliated to PL38 family, Oal17A1 and Oal17A2 were classified as PL17 family members, and the other eight alginate lyases all were identified as PL7 family proteins. The alginate lyase with the largest molecular weight (Mw) was Aly6A (95.1 kDa) and the smallest was Aly7A3 (32.2 kDa). The theoretical isoelectric points (pI) of 12 alginate lyases were in the range of 4.0–6.0, with the exception of Aly38A and Aly7A3, which tended to be neutral. Only Aly38A and Oal17A1 were predicted to be unstable proteins (instability coefficient > 40). According to the prediction, grand average of hydropathicity (GRAVY) values of all alginate lyases were less than 0, which indicated these proteins were hydrophilic proteins.

Among these 12 alginate lyases, except Aly7A1 and Aly7A4, their amino acid sequences were obtained the most similar sequence in the NCBI Reference proteins database, with more than 97% similarity. Meanwhile, all of these most similar sequences were from the strains of the *Agarivorans* genus (Table 1). For example, Aly7A2 showed 98.55% amino acid sequence similarity with WP_221076907 from *A. aestuarii*, and Aly7B1 exhibited the highest similarity of 99.81% with WP_016400966 from *A. albus*. In summary, five alginate lyases were similar with the sequences from *A. albus*, four alginate lyases similar with the sequences from *A. aestuarii*, and one alginate lyase similar with the sequence from *A. litoreus.* Therefore, these 10 alginate lyases might be common within this genus. However, Aly7A1 matched the alginate lyase sequence WP_164648089 from *Vibrio astriarenae* with the highest similarity of 55.38%, and Aly7A4 also matched the same sequence with the highest similarity of 54.75%, suggesting these two alginate lyase coding genes might be gained from *Vibrio* strains through horizontal gene transfer. *Vibrio astriarenae* sp. nov. had been documented as an agarolytic marine bacterium [35,36], and several alginate lyases could be annotated in the genome. Remarkably, these two alginate lyases, Aly7A1 and Aly7A4, only had eight base differences in their gene sequences, producing five amino acid differences in the amino acid composition. Such small differences of Aly7A1 and Aly7A4 might result in the different protein forms having different properties, while the predicted physical and chemical characters were very similar.

### 2.5. Conserved Domain and Protein Structure Prediction of Alginate Lyases

**Figure 4:**
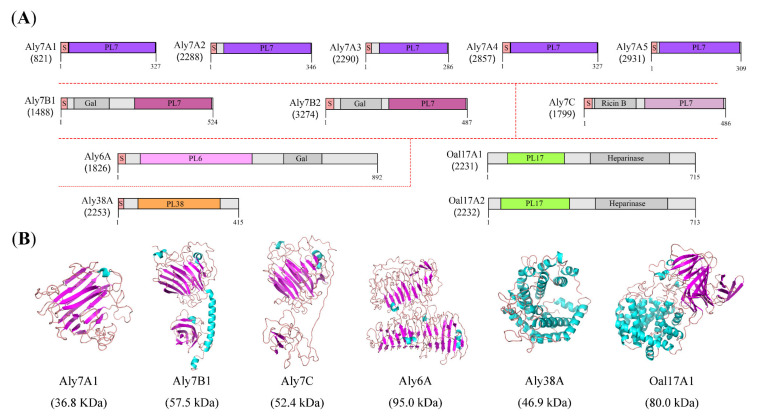
Conserved domains and structure representations of alginate lyase from *Agarivorans* sp. B2Z047. (**A**) Domain architectures of all alginate lyases. Aly7A proteins (Aly7A1, Aly7A2, Aly7A3, Aly7A4, and Aly7A5) and Aly38A contained the signal peptide and the catalytic domain. Aly7B proteins (Aly7B1 and Aly7B2), Aly7C, and Aly6A harbored the catalytic domain and a potential carbohydrate-binding domain. Oal17A1 and Oal17A2 had two catalytic domains: alginate lyase and heparinase. S, signal peptide; Gal, Galactose-binding-like domain (IPR008979); Ricin B, Ricin B-like lectins (IPR035992); and Heparinase, Heparinase II/III-like domain (IPR012480). (**B**) Overall structure of alginate lyases. Aly7A1, Aly7B1, and Aly7C, β-sandwich jelly roll; Aly6A, β-helix fold; Aly38A, (α/α)_7_ barrel; Oal17A1, (α/α)_6_ toroid + anti-parallel β-sheet. All the structures were representatives of each alginate lyase type of strain B2Z047 and shown as cartoon. α-helixes are shown in cyan and β-sheets are shown in purple.

The amino acid sequences of these 12 alginate lyases were submitted to the InterPro (http://www.ebi.ac.uk/interpro/) to analyze their domain architectures. The results showed that ten of them had a signal peptide in the N-terminus (Figure 4A), which suggested that they might be secreted and functioned outside of the cell or in the periplasmic. Both Oal17A1 and Oal17A2 were deficient in the signal peptide and might be located in the cytoplasm. For signal peptide containing alginate lyases, the LipoP-1.0 server was used to identify the lipoprotein signal peptides. The predictions indicated that Aly7B1, Aly7B2, and Aly6A possessed the SpII lipoprotein signal peptides, while the remaining alginate lyases held the SpI lipoprotein signal peptides. The lipoproteins crossed the inner membrane by the general secretory system (Sec), and then anchored to the inner membrane by its N-terminal signal peptide for next maturation [37]. In the model bacterium *Escherichia coli*, most lipoproteins were transferred to the outer membrane by the Lol system, unless the residue at position +2 was an aspartate (Asp), which would remain in the inner membrane. The residues at position +2 of Aly7B1, Aly7B2, and Aly6A were the threonine (Thr), glycine (Gly), and asparagine (Asn), respectively. Thus, these three alginate lyases might be targeted to the outer membrane. Following, the subcellular localization of all alginate lyases were predicted by the BUSCA webserver, and the results showed that Oal17A1 and Oal17A2 were in the cytoplasm, Aly7C was on the plasma membrane for a transmembrane α-helix, and the remaining proteins were in the extracellular space. Given the above prediction results, the subcellular localization of all alginate lyases was proposed as Oal17A1 and Oal17A2 in the cytoplasm, Aly7C on the plasma membrane, Aly7B1, Aly7B2, and Aly6A on the outer membrane, and Aly7A proteins (Aly7A1, Aly7A2, Aly7A3, Aly7A4, and Aly7A5) and Aly38A outside the cell.

The InterPro protein domain analyses also showed that, except for the signal peptide, Aly7A proteins consisted of a PL7 domain, and Aly38A only contained a PL38 domain (Figure 4A). Six other alginate lyases were composed of two conserved domains. Aly7B proteins (Aly7B1 and Aly7B2) and Aly7C harbored the C-terminal PL7 domain, but the N-terminal domains were different. Aly7B proteins contained the Galactose-binding-like domain (IPR008979) in the N-terminus, which had been found in several different proteins, and the common function was to bind to specific ligands of carbohydrate substrates. Aly7C possessed the N-terminal Ricin B-like lectins domain (IPR035992), which was present in many carbohydrate-recognition proteins and had been shown to bind sugars. Unlike PL7 proteins, Aly6A harbored the N-terminal PL6 domain and the C-terminal Galactose-binding-like domain (IPR008979). In addition, Oal17A proteins (Oal17A1 and Oal17A2) were composed of the N-terminal PL17 domain and the C-terminal Heparinase II/III-like domain (IPR012480). Proteins containing a Heparinase II/III-like domain were found to be oligosaccharide lyases and function in the degradation of alginate. These domains might be helpful to improve the binding efficiency of enzyme molecules and substrates [30]. Therefore, based on the domain architectures, the 12 alginate lyases of strain B2Z047 could be divided into six types: Aly7A, Aly7B, Aly7C, Aly6A, Aly38A, and Oal17A proteins.

The 3D models of alginate lyase were constructed by Iterative Threading ASSEmbly Refinement (I-TASSER), and pairwise structural similarity was measured with TM-align based on the TM-score (Table S2). The overall structure of Aly7A proteins, and the PL7 domains of Aly7B and Aly7C proteins were predicted to fold as a β-sandwich jelly roll, which was the typical PL7 family structure (Figure 4B). In Aly7B proteins, an α-helix domain linked the PL7 domain and the Galactose-binding-like domain. In Aly7C protein, the PL7 domain was connected with the Ricin B-like domain by a flexible linker. The linker region universally occurred in carbohydrate-degrading enzymes with different lengths and compositions, promoting the functional interactions between structured domains [38,39]. Aly6A presented the classical parallel β-helix structure of PL6 family proteins, while the Galactose-binding-like domain also showed parallel β-helix fold. Aly38A was a typical structure of (α/α)_7_ barrel, which was consistent with the PL38 family member. Until now, alginate lyase activity of PL38 family protein was not determined experimentally, though some PL38 family proteins had been identified as alginate lyases by the NCBI Prokaryotic Genome Annotation Pipeline.

Both Oal17A1 and Oal17A2 had the closest structure similarity to AlyA3 (PDB code: 7BJT) with the TM-score of 0.981 and 0.990, respectively, which belonged to PL17 family [9]. For these two Oal17A proteins, each monomer consisted of two domains: α-toroid and β-sheet, and the homodimer eventually formed and functioned. Given the enzyme activity of all the characterized PL17 proteins displaying in CAZy database, except alginate lyase (Aly II), Oal17A1 and Oal17A2 were the potential oligoalginate lyases, degrading oligoalginates to the monomer, 4-deoxy-L-erythro-5-hexoseulose uronic acid (DEH) and being a critical step for the utilization of alginate.

### 2.6. Enzymatic Activity of Alginate Lyases from Strain B2Z047

**Figure 5:**
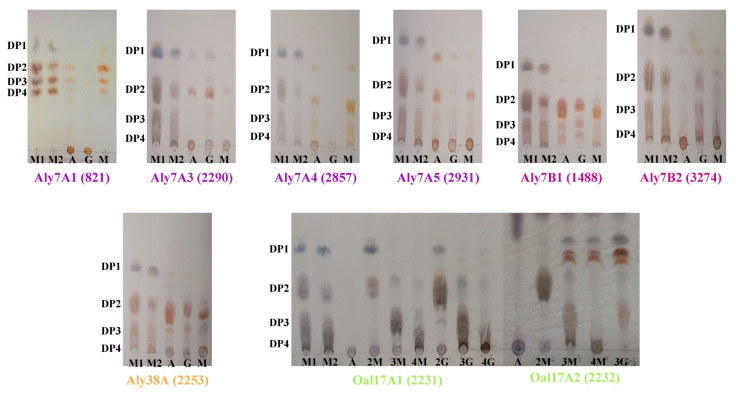
The degradation products of the alginate lyases for 2 h reaction. Lane M1, oligosaccharides standards from β-d-mannuronic acids; Lane M2, oligosaccharides standards from α-l-guluronic acids; DP1, DP2, DP3, and DP4 represented mono-, di-, tri-, and tetrasaccharides, respectively. Lane A, degradation products of sodium alginate; Lane G, degradation products of polyG; Lane M, degradation products of polyM. Lane 2M, 3M, and 4M represented the degradation results of di-, tri-, and tetramannuronic acids, respectively. Lane 2G, 3G, and 4G represented the degradation results of di-, tri-, and tetraguluronic acids, respectively. These figures were representatives of triplicate experiments.

To further characterize the function of these potential alginate lyases, all genes were cloned from the genome of *Agarivorans* sp. B2Z047 and overexpressed in *E. coli* BL21 (DE3) without the signal peptide. Ultimately, the soluble expressions of eight alginate lyases were obtained, and the enzymatic activities were detected from the crude supernatant of the host bacteria. Considering that Oal17A1 and Oal17A2 might be oligosaccharide lyases, we tested the degradation products with the thin-layer chromatography (TLC) method (Figure 5). Aly7A3, Aly7A5, Aly7B1, Aly7B2, and Aly38A could degrade sodium alginate, polyM and polyG. Aly7A3 degraded them into mono- and disaccharides. Aly7A5 degraded the substrates into mono-, di-, and trisaccharides, whereas the products from polyM and polyG were different. Aly7B1 degraded the substrates into di-, and trisaccharides, and some products migrating below the standard trimers. Aly7B2 degraded the substrates into mono-, di-, tri-, and tetrasaccharides. The dominant degradation products of Aly38A were disaccharides. There were five amino acid differences between Aly7A1 and Aly7A4, and both of them only degraded polyM and sodium alginate, but did not act on polyG. PolyM and sodium alginate were degraded into mono-, di-, tri-, and tetrasaccharides by Aly7A1 and Aly7A4. Through the TLC detection, it was found that Oal17A1 could degrade disaccharides into monosaccharide, but had no degrading ability for tri-, and tetrasaccharides. Furthermore, Oal17A2 degraded tri- and tetrasaccharides into monosaccharide, and some products migrating above the standard monomers. Both oligomannuronic acids and oligoguluronic acids could be depolymerized by Oal17A1 and Oal17A2. These activity analyses confirmed the above functional prediction, and further explained the biotransformation of alginates, containing polyM and polyG, to oligosaccharides, and then to monosaccharide by the combination of alginate lyases of strain B2Z047.

### 2.7. Identification of Key Alginate Lyases

**Figure 6:**
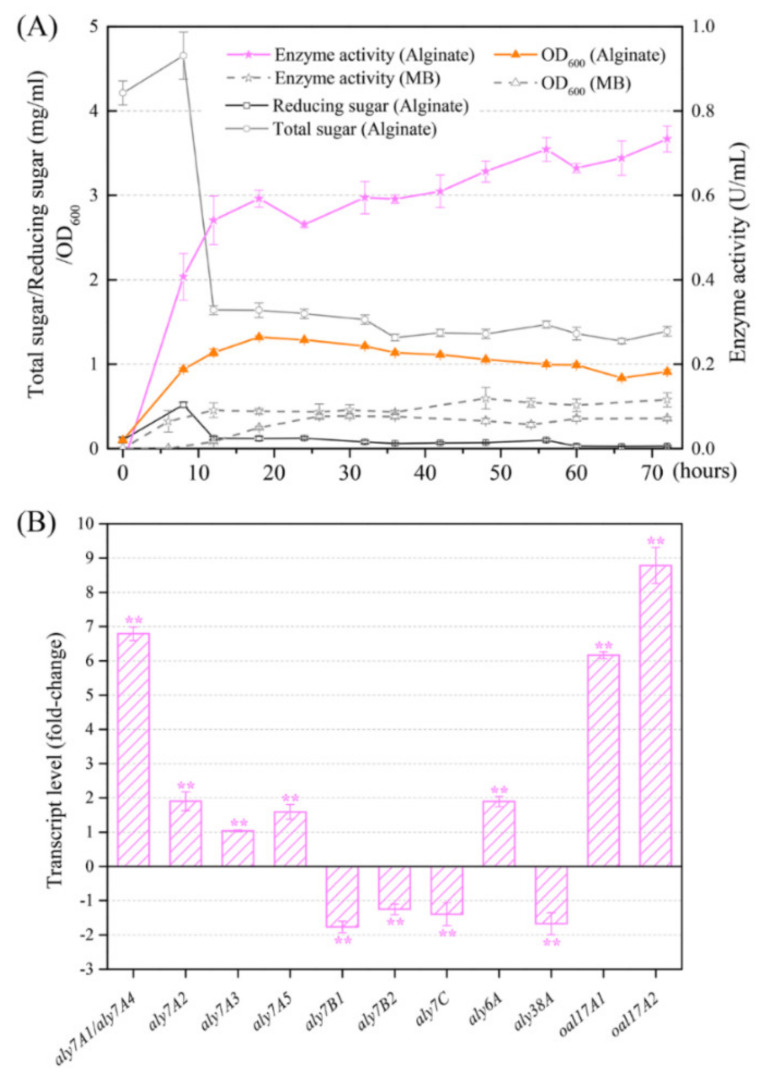
Secretion and expression of alginate lyases of strain B2Z047. (**A**) Growth, enzyme activity, reducing sugar, and total sugar curves of strain B2Z047 in the MB medium (MB) and the alginate containing medium (Alginate). (**B**) Expression level of alginate lyase genes in strain B2Z047 after 12 h incubation. The sample from the MB medium was set as the reference (Transcript level was 1). The error bars represent the standard deviation, and asterisks denote *p*-values for *t*-tests of differences from 1: ** *p* < 0.01.

In order to understand the combination of all alginate lyases functioning in the alginate-degrading process, growth curve, and alginate lyase production of strain B2Z047 cultivated with or without sodium alginate were detected at different time points (Figure 6A). When cultivated in the alginate containing medium, strain B2Z047 exhibited a typical bacterial growth curve with exponential and stationary phases. MB medium was not effective in the growth of strain B2Z047 due to the slow rise of cell density, which was far lower than that of alginate containing medium. The alginate lyase activity in the supernatant of strain B2Z047 culturing in the alginate containing medium increased rapidly from 0 h to 12 h, and had almost reached the summit. Although the alginate lyase activity subsequently increased, it tended to be flat after 12 h. Meanwhile, the alginate lyase activity of the MB culture was always at the low level. Thus, the presence of alginate was beneficial to the growth and alginate lyase production of strain B2Z047.

Considering the production of alginate lyase during this process, alginate could be degraded by alginate lyase and the reducing sugar was released. Therefore, alginate might reduce and the products might increase. If strain B2Z047 could not use alginate or its products as carbon sources, the content of total sugar would be constant, and the content of reducing sugar would increase. If strain B2Z047 utilized alginate or its products, the content of total sugar and reducing sugar might fall. To determine the alginate utilization of strain B2Z047, the contents of total sugar and reducing sugar were measured individually. The content of total sugar was determined by the phenol–sulfuric acid method [40]. In this method, sulfuric acid broke down any polysaccharides, oligosaccharides, and disaccharides to monosaccharides, and thus all classes of carbohydrates, alginate, and its products, could be detected. The contents of total sugar and reducing sugar in the cell-free supernatant of the alginate adding cultures were measured. The total sugar content was initially high and increased slowly within the first 8 h of cultivation, but dropped dramatically from 8 to 12 h, which appeared to result from the rapid growth and the sugar consumption of strain B2Z047 during this period. Correspondingly, the reducing sugar content increased from 0 to 8 h, and then declined after 8 h. When cultured in the alginate adding medium, strain B2Z047 might be induced and produce plenty of alginate lyases in the initial stage (between 0 and 8 h), and then degrade sodium alginate into oligo- or monosaccharide. Thus, total sugar and reducing sugar increased during this period. The oligo- or monosaccharide might be further utilized and consumed by strain B2Z047 for growth between 8 and 12 h. During the process, some oligosaccharides might be transferred into cells and then degraded into monosaccharide. The production and the consumption of oligosaccharides might cause the content changes of total sugar and reducing sugar. Moreover, cell density increased between 0 and 8 h, but the total sugar was also added during this period, which might be because yeast extract was responsible for the initial bacterial growth and insoluble sodium alginate was dissolved. Therefore, alginate lyases were supposed to be induced and produced by strain B2Z047 during the alginate-degrading process.

To better analyze the alginate lyases differences of strain B2Z047 in the medium supplemented with or without sodium alginate and to identify the alginate lyases playing key roles in the degradation of alginate, the real-time fluorescence quantitative PCR (q-PCR) was applied. The 12 h transcript level of alginate lyase genes was determined, and the transcript level of 16S rRNA gene was used as control. There was only eight bases difference between the gene sequences of *Aly7A1*(*821*) and *Aly7A4*(*2857*), and these bases were distributed throughout the gene. Thus, the specific primers could not be designed and the expression level of these two genes could not be discriminated by q-PCR, resulting in the *aly7A1/aly7A4* data representing the combined effect of these two genes. The expression levels of *aly7A1*/*aly7A4*, *aly7A2*, *aly7A3*, *aly7A5*, *aly6A*, *oal17A1*, and *oal17A2* were significantly up-regulated, whereas the remaining four alginate lyase coding genes were expressed at the lower level in the medium supplemented with sodium alginate (Figure 6B). These up-regulated genes might be induced and play the most crucial role in the degradation of alginate. Among them, Aly7A proteins (Aly7A1, Aly7A2, Aly7A3, Aly7A4, and Aly7A5) and Aly6A might degrade alginate to oligosaccharides, and then Oal17A1 and Oal17A2 could degrade oligosaccharides to monosaccharide. Significantly, *oal17A2* displayed the highest increase (8.78 times), followed by *aly7A1*/*aly7A4* (6.79 times) and *oal17A1* (6.16 times). As *alyA1* and *alyA4* could not be discriminated by q-PCR, it was still difficult to determine which one was induced, or if both could be induced. The potential enzyme activity differences of Aly7A1 and Aly7A4 deserve to be explored further, which might provide a special material to study the function of the specific amino acid residues.

### 2.8. Potential Alginate-Degrading Pathway of Strain B2Z047

**Figure 7:**
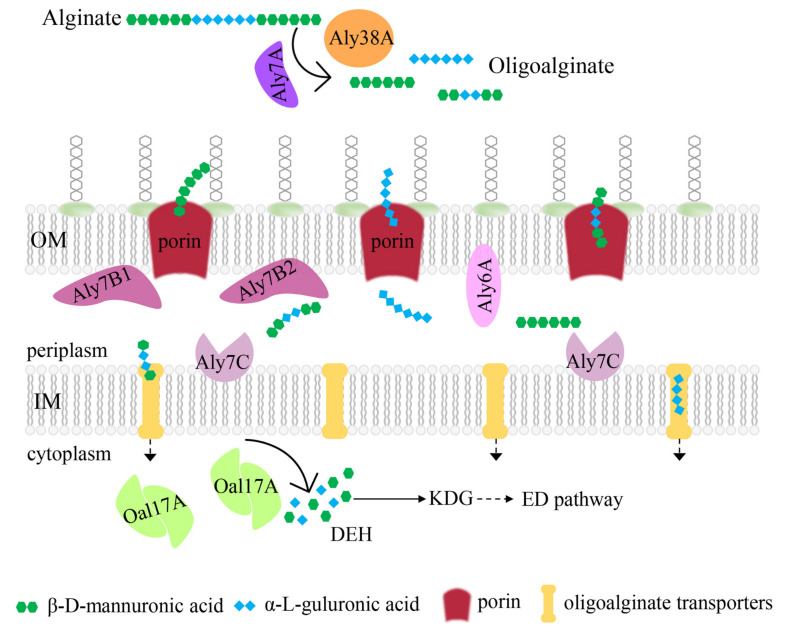
Proposed model of *Agarivorans* sp. B2Z047 for alginate utilization. Based on the signal peptide, the lipoprotein signal peptide and the subcellular localization predictions, all 12 alginate lyases were displayed in the specific location of the bacterial cell. The enzymatic activity of each alginate lyase was from the function prediction and the degradation products assay of alginate lyase overexpressed in *E. coli* BL21 (DE3). Porin (KdgMN), oligoalginate transporters, DEH reductase (DehR) and ED pathway were annotated in the genome of strain B2Z047 (Table S3). The abbreviations are as follows: Aly7A proteins, Aly7A1, Aly7A2, Aly7A3, Aly7A4, and Aly7A5; Oal17A proteins, Oal17A1 and Oal17A2; DEH, 4-deoxy-L-erythro-5-hexoseulose uronic acid; KDG, 2-keto-3-deoxy-gluconate; ED pathway, Entner–Doudoroff pathway.

To date, three different strategies have been reported for alginate-utilizing bacteria: the polysaccharide utilization loci (PUL) system, the “scattered” system, and the “pit” transport system [11]. In this study, *Agarivorans* sp. B2Z047 was screened as a highly efficient alginate-degrading bacterium. When cultivated in the medium containing 1.1% sodium alginate, 0.3% yeast extract, 1% NaCl, and 0.1% MgSO_4_, strain B2Z047 was prone to produce more alginate lyases. All 12 alginate lyase genes were scattered in the genome of strain B2Z047, and the majority were similar to the alginate lyase genes of three other *Agarivorans* strains. The amino acid sequence predictions showed Oal17A1 and Oal17A2 in the cytoplasm, Aly7C on the plasma membrane, Aly7B1, Aly7B2, and Aly6A on the outer membrane, and Aly7A proteins (Aly7A1, Aly7A2, Aly7A3, Aly7A4, and Aly7A5) and Aly38A outside the cell. The domain architectures, protein structures, and enzymatic activity analyses further illustrate the function of these alginate lyases. Additionally, the transcript levels of these genes identified the key alginate lyases functioning in the alginate-degrading process. Therefore, strain B2Z047 could utilize alginate completely, and a simplified model was proposed (Figure 7).

Outside the cell, alginate was first degraded into oligoalginates by Aly7A proteins (Aly7A1, Aly7A2, Aly7A3, Aly7A4, and Aly7A5) and Aly38A. The oligomers were then translocated through the outer-membrane porin (KdgMN) into the periplasm, where those oligomers were degraded into di-, tri-, and tetrasaccharides by periplasmic alginate lyases (Aly6A, Aly7B, and Aly7C proteins). These subsequent oligomers were transported into the cytoplasm via oligoalginate transporters. Oligoalginate lyases Oal17A then degraded oligomers into monomer units. All of the produced monosaccharide were non-enzymatically converted to 4-deoxy-L-erythro-5-hexoseulose uronic acid (DEH). DEH was converted by DEH reductase (DehR) to 2-keto-3-deoxy-gluconate (KDG), which entered the ED pathway. These predicted results indicated that strain B2Z047 had a “scattered” system to utilize alginate.

## 3. Materials and Methods

### 3.1. Alginate-Degrading Strains Isolated

Firstly, 1 g abalone gut was serially diluted to 10^−6^ with sterilized artificial seawater (3% NaCl, 0.23% MgCl_2_, 0.32% MgSO_4_, 0.12% CaCl_2_, 0.07% KCl, and 0.02% NaHCO_3_, *w*/*v*), and 100 µL aliquots of each dilution were spread on marine agar 2216 (BD, Franklin Lakes, MA, USA. The plates were incubated at 25 °C for 7 days, and then the colonies were isolated on the basis of morphological differences. All strains were transferred to the fresh MA medium for strain cultivation and preservation. Then, the alginate-degrading strains were screened on the modified MA medium supplemented with 0.5% sodium alginate. After strains were grown at 25 °C for 48 h, 10% CaCl_2_ was poured onto the plates and soaked the colonies for 30 min to observe whether transparent circles appeared around the colonies. The diameter of the transparent ring was measured and recorded. The strains holding larger transparent circles were cultivated in marine broth 2216 (MB; BD, Franklin Lakes, USA) supplemented with 0.5% sodium alginate. After 24 h of incubation, the culture of each strain was centrifuged at 8000 rpm for 5 min and the supernatant was filtered by a 0.22-μm filter to remove the cells. The cell-free supernatant of each strain was used to determine the alginate lyase activity.

### 3.2. Enzyme Activity Assay

The enzyme activity of the alginate lyase was determined by measuring the release of reducing sugar using the 3,5-dinitrosalicylic acid (DNS) colorimetry [29]. Standard curves were gained using glucose ranging from 0 to 1 mg/mL. A total of 200 μL DNS solution was mixed with 300 μL degradation product of alginate lyase, immersed in a boiling water bath for 7 min and then immediately cooled to room temperature. Next, 500 µL ddH_2_O was added and mixed completely. An aliquot of 200 µL mixture was transferred into a 96-well clear plastic plate. The absorbance of each sample well was measured at 540 nm. One unit (U) of enzyme activity was defined as the amount of enzyme required to release 1 µmol of reducing sugar per min.

During the alginate-degrading strain screening, the alginate lyase reaction was performed at 40 °C for 20 min in a mixture of 100 µL sodium alginate (5 mg/mL), 100 µL PBS (pH 7.4), and 100 µL cell free supernatant of each strain in triplicate. Then, 100 µL of DNS solution was added and the mixture was boiled for 5 min to terminate the reaction. For strain B2Z047, the enzymatic reaction condition was optimized. The alginate lyase reaction was carried out at 40 °C for 15 min in a mixture of 200 µL sodium alginate (5 mg/mL) and 100 µL cell free supernatant in triplicate. After incubation, 200 µL of DNS solution was added. Then, the mixture was boiled for 7 min to terminate the reaction and 500 µL ddH_2_O was supplemented. Finally, 200 µL reaction mixture was pipetted to 96-well plates and the absorbance was measured at 540 nm.

### 3.3. Optimization of Alginate Lyase Production Conditions

When the carbon sources were tested, 0.5% carbon source, containing sodium alginate, agar, carrageenan, starch, saccharose, cellulose, glucose, and Na_2_CO_3_, was individually supplemented into the medium containing 0.5% peptone in artificial seawater (pH 7.0). When the nitrogen sources were investigated, tryptone (0.5%), yeast extract (0.5%), beef extract powder (0.5%), fish peptone (0.5%), (NH_4_)_2_SO_4_ (0.28%), NaNO_3_ (0.36%), and urea (0.06%) was separately added into the medium containing 0.5% sodium alginate in artificial seawater (pH 7.0). The routine culture condition was set as follows: 50 mL liquid medium in a 250 mL flask (1/5), at 28 °C and 150 rpm. Each of these factors was changed one by one when the optimization experiment was carried out. These assays were performed with three biological replicates each time and repeated more than three times.

### 3.4. Whole Genome Sequencing, Assembly, and Annotation

Cells of strain B2Z047 in exponential phase were collected, and the genomic DNA was extracted and sequenced on PacBio platform (GENEWIZ, Suzhou, China). The G+C content of the chromosomal DNA was calculated using genome sequence. The draft genome sequence of strain B2Z047 were sequenced on the Illumina HiSeq PE150 platform (Novogene, Beijing, China). The genome sequences of strain B2Z047 were deposited in the DDBJ/GenBank/EMBL database. Other genome sequences were obtained from NCBI. The average nucleotide identity (ANI) was calculated using ANI Calculator (https://www.ezbiocloud.net/tools/ani, [41]). The digital DNA–DNA hybridization (dDDH) was calculated using the Genome-to-Genome Distance Calculator 3.0 (http://ggdc.dsmz.de/ggdc.php, [42]).

### 3.5. Phenotypic Characteristics

The morphological and physiological features of strain B2Z047 were examined after incubation at 30 °C for 48 h on MA plates. Cell morphology and size were observed by transmission electron microscope (JEM-1100; JEOL, Tokyo, Japan). Gram reaction was determined using the method described by Smibert and Krieg [43]. Motility was determined by the hanging-drop method and gliding motility was tested by inoculating the bacteria on the 0.3% agar as described by Bowman [44]. Growth range and optimum temperature were evaluated by observation of visible colonies on MA plates at various temperatures (0, 4, 10, 15, 25, 28, 30, 33, 35, and 37 °C). The effects of different salt concentrations on growth were tested using a medium comprised of 0.1% yeast extract and 0.5% peptone, prepared with artificial seawater and in the presence of 0–10% (*w*/*v*) NaCl at intervals of 1%. The effects of pH were determined by adding the appropriate buffers (20 mM), including MES (pH 5.5 and 6.0), PIPES (pH 6.5 and 7.0), HEPES (pH 7.5 and 8.0), Tricine (pH 8.5), and CAPSO (pH 9.0 and 9.5) to MB media, separately. OD_600_ values of the cultures were measured after incubation for two days at 30 °C. Growth under anaerobic condition was examined after 2 weeks of incubation on MA plates in an anaerobic jar with or without 0.1% (*w*/*v*) KNO_3_. Catalase activity was detected by bubble production in 3% (*v*/*v*) H_2_O_2_. Oxidase activity was determined using an oxidase reagent kit (bioMérieux, Craponne, France) according to the manufacturer’s instruction. Bacterial abilities to hydrolyse agar, DNA, alginate, starch, casein, CM-cellulose, and Tweens (20, 40, 60, and 80) were tested according to the methods from Dong and Cai [45].

### 3.6. 16S rRNA Gene Sequence Analyses

Using Taq DNA polymerase and universal primers 27F and 1492R, the 16S rRNA gene sequence was amplified. The purified PCR product with 3’-A overhangs was cloned into a pMD18-T vector (Takara), and the ligation product was transformed into *E. coli* DH5α cells. The positive clones were selected and sequenced. The 16S rRNA gene sequences of 100 strains with alginate-degrading activity were aligned using muscle [46]. The multiple sequence alignment included automated removal of spurious sequences and poorly aligned regions using trimAl [47]. The phylogenetic trees were reconstructed using IQTree [48] using GTR+F+I+G4 and visualized using ITOL [49]. Bootstrap values were evaluated based on 1000 replicates.

A near-complete 16S rRNA gene sequence (1545 bp) of strain B2Z047 was obtained and submitted to the GenBank database. The 16S rRNA gene similarities were calculated using the EzBioCloud Database [50]. Multiple sequences were aligned using the CLUSTAL_X program [51]. In order to determine the phylogenetic position of strain B2Z047, phylogenetic trees were reconstructed by the neighbor-joining [52], maximum-parsimony [53], and maximum-likelihood [54] methods in MEGA 7.0 [55]. Evolutionary distances were calculated according to the algorithm of Kimura’s two-parameter model [56] for the maximum-likelihood method. Bootstrap values were determined based on 1000 replications.

### 3.7. Alginate Lyase Sequence Analyses

The putative alginate lyases and other carbohydrate-active enzymes were identified by the CAZy database using the dbCAN2 meta server (https://bcb.unl.edu/dbCAN2/, [57,58]). The amino acid residues encoding a putative signal peptide were predicted by the SignaIP 5.0 server (http://www.cbs.dtu.dk/services/SignalP/). Molecular weight (MW), theoretical isoelectric point (pI), instability index, and grand average of hydropathicity (GRAVY) were estimated using the peptide mass tool on the ExPASy server (https://web.expasy.org/protparam/). Domain architecture was predicted by InterPro (http://www.ebi.ac.uk/interpro/). The three-dimensional structure of alginate lyase was modelled with I-TASSER (https://zhanggroup.org/I-TASSER/, [59]). Structural similarity of two protein structures was measured with TM-align based on the TM-scores [60], varying from 0 to 1. The higher the TM-score, the more similar are the two aligned structures. Proteins involved in alginate metabolism were annotated through the COG and dbCAN-PUL (https://bcb.unl.edu/dbCAN_PUL/) database. The LipoP-1.0 server (https://services.healthtech.dtu.dk/service.php?LipoP-1.0, [61]) was used to identify the lipoprotein signal peptides, and the BUSCA webserver was used for predicting protein subcellular localization (http://busca.biocomp.unibo.it, [62]).

### 3.8. Cloning and Expression of Alginate Lyase and Its Degradation Products

*E. coli* DH5α and the vector pET28a were used for gene cloning. *E. coli* BL21 (DE3) and the vector pET28a were used for protein expression. The alginate lyase gene sequence was amplified using the specific primers (Table S4). The recombinant cells were grown in LB medium supplemented with kanamycin (40 µg/mL) at 37 °C and 175 rpm until the optical density at 600 nm reached 0.6–0.8, and then the recombinant protein was induced by the addition of 0.1 mM of IPTG at 16 °C for 20 h. The cells were collected by centrifugation at 8000 rpm for 10 min and then washed with Tris-NaCl buffer (20 mM Tris, 50 mM NaCl, pH 7.4). The cells were resuspended in Tris-NaCl buffer and lysed by sonication on an ice slurry, and then the suspension was centrifuged at 12,000 rpm for 30 min to remove intact cells and debris. The protein solutions were tested by SDS-PAGE and Coomassie blue staining for three biological replicates.

To determine the oligosaccharide compositions of the final digests, 100 µL enzyme was mixed with 400 µL substrate (0.5%, *w*/*v*). After incubation at 40 °C for 2 h, the reaction was terminated by boiling for 10 min. After centrifugation at 8000 rpm for 5 min, 6 μL of supernatant was analyzed by pro-coated silica gel 60 F254 TLC plates (Merck, Darmstadt, Germany) with a mixture of 1-butanol/acetic acid/water (2:1:1, *v*/*v*/*v*) as a developing system. Staining was performed using the agent containing 1 mg/mL 1,3-dihydroxynaphthalene, 10% concentrated sulfuric acid and 50% ethanol, followed by incubation at 110 °C for 10 min. PolyM, polyG (6–8 kDa), and oligosaccharide substrates were purchased from Zzstandard (Shanghai, China).

### 3.9. Total Sugar Assay

The total sugar was determined by the phenol–sulfuric acid method at 490 nm. Standard curves were made with alginate ranging from 0 to 5 mg/mL. 1 mL 6% phenol and 3 mL sulfuric acid was added to 100 μL samples. The mixture was cooled for 30 min. An aliquot of 200 µL of each sample was transferred into a 96-well clear plastic plate. The absorbance of each sample well was then measured at 490 nm.

### 3.10. q-PCR Assay Based on SYBR Green

Alginate lyase gene expression level was detected by q-PCR with SYBR Green method. Strain B2Z047 was grown 12 h in the MB medium and the alginate containing medium, then the samples were harvested. RNA was isolated with the BIOZOL-total RNA Extraction reagent using the manufacturer’s suggested protocol. cDNA was generated using ABScript II RT Master Mix for q-PCR with gDNA Remover (ABclonal, Wuhan, China) by following the manufacturer’s protocol. q-PCR was performed in triplicate using the primers listed in Table S4. The cycle threshold (Ct) value was determined by StepOnePlus™ Real-Time PCR System (Thermo Fisher Scientific, Waltham, MA, USA). The q-PCR reactions were performed in a 20 μL mixture containing 10 μL of 2×Universal SYBR Green Fast qPCR Mix (ABclonal, Wuhan, China), 0.4 μL of each primer, 1 μL DNA template, and 8.6 μL ddH_2_O. The q-PCR parameters were 95 °C for 180 s, followed by 40 cycles at 95 °C for 5 s, and 60 °C for 30 s.

## Figures and Tables

**Table 1:** Information of 12 alginate lyases.

Gene Locus	Protein Name	Length (aa)	Mw (kDa)	pI	Instability Index	GRAVY	Most Similar Sequence		PL Family
							Sequence ID (%)	Organism	
821	Aly7A1	327	36.7	4.66	30.51	−0.420	WP_164648089 (55.38)	*Vibrio astriarenae*	PL7
2288	Aly7A2	346	38.3	5.93	19.83	−0.522	WP_221076907 (98.55)	*Agarivorans aestuarii*	PL7
2290	Aly7A3	286	32.2	6.53	25.97	−0.535	WP_040307007 (98.60)	*Agarivorans albus*	PL7
2857	Aly7A4	327	36.8	4.63	29.92	−0.422	WP_164648089 (54.75)	*Vibrio astriarenae*	PL7
2931	Aly7A5	309	34.4	5.93	23.80	−0.570	WP_220718128 (98.71)	*Agarivorans litoreus*	PL7
1488	Aly7B1	524	57.5	4.99	35.73	−0.469	WP_016400966 (99.81)	*Agarivorans albus*	PL7
3274	Aly7B2	487	53.1	4.26	37.40	−0.560	WP_221074174 (97.95)	*Agarivorans aestuarii*	PL7
1799	Aly7C	486	52.4	5.26	33.12	−0.573	WP_221077217 (99.43)	*Agarivorans aestuarii*	PL7
1826	Aly6A	892	95.1	4.16	31.75	−0.291	WP_221076585 (98.43)	*Agarivorans aestuarii*	PL6
2253	Aly38A	415	46.9	6.99	40.25	−0.578	WP_016404079 (99.04)	*Agarivorans albus*	PL38
2231	Oal17A1	715	80.0	5.45	41.05	−0.302	WP_016403996 (99.58)	*Agarivorans albus*	PL17
2232	Oal17A2	712	80.1	5.40	35.63	−0.388	WP_016403995 (99.72)	*Agarivorans albus*	PL17

## Data Availability

The GenBank accession number for the 16S rRNA gene sequence of strain B2Z047 is OM278383. The draft genome and the complete genome of strain B2Z047 have been deposited at DDBJ/ENA/GenBank under the accession number WHRY00000000 and CP088080, respectively.

## Supplementary Materials

The following supporting information can be downloaded at: https://www.mdpi.com/article/10.3390/md20040254/s1, Data S1: Information of 100 alginate-degrading strains. Figure S1: Optimization of alginate lyase production conditions of strain B2Z047; Figure S2: The colony morphology of strain B2Z047 grew on MA plate at 30 °C for two days; Table S1: Four factors and three levels of orthogonal experiment; Table S2: Function of the closest structure and structural similarity (TM-score) of alginate lyases; Table S3: Genes for the alginate-degrading process in the genome of *Agarivorans* sp. B2Z047; Table S4: Primers used in this study.

## References

[1] Field C.B., Behrenfeld M.J., Randerson J.T., Falkowski P. (1998). Primary production of the biosphere: Integrating terrestrial and oceanic components. Science.

[2] Haug A., Larsen B., Smidsrod O. (1967). Studies on sequence of uronic acid residues in alginic acid. Acta Chem. Scand..

[3] Strand B.L., Morch Y.A., Skjak-Braek G. (2000). Alginate as immobilization matrix for cells. Minerva Biotecnol..

[4] Liu J., Yang S., Li X., Yan Q., Reaney M.J.T., Jiang Z. (2019). Alginate oligosaccharides: Production, biological activities, and potential applications. Compr. Rev. Food Sci. Food Saf..

[5] Zhu B., Yin H. (2015). Alginate lyase: Review of major sources and classification, properties, structure-function analysis and applications. Bioengineered.

[6] Cheng D., Jiang C., Xu J., Liu Z., Mao X. (2020). Characteristics and applications of alginate lyases: A review. Int. J. Biol. Macromol..

[7] Yu Z.C., Zhu B.W., Wang W.X., Tan H.D., Yin H. (2018). Characterization of a new oligoalginate lyase from marine bacterium *Vibrio* sp.. Int. J. Biol. Macromol..

[8] Li S.Y., Wang L.N., Chen X.H., Zhao W.W., Sun M., Han Y.T. (2018). Cloning, expression, and biochemical characterization of two new oligoalginate lyases with synergistic degradation capability. Mar. Biotechnol..

[9] Park D., Jagtap S., Nair S.K. (2014). Structure of a PL17 family alginate lyase demonstrates functional similarities among exotype depolymerases. J. Biol. Chem..

[10] Ochiai A., Yamasaki M., Mikami B., Hashimoto W., Murata K. (2010). Crystal structure of exotype alginate lyase Atu3025 from *Agrobacterium tumefaciens*. J. Biol. Chem..

[11] Zhang L., Li X., Zhang X., Li Y., Wang L. (2021). Bacterial alginate metabolism: An important pathway for bioconversion of brown algae. Biotechnol. Biofuels.

[12] Wang B., Dong S., Li F.L., Ma X.Q. (2021). Structural basis for the exolytic activity of polysaccharide lyase family 6 alginate lyase BcAlyPL6 from human gut microbe *Bacteroides clarus*. Biochem. Biophys. Res. Commun..

[13] Lyu Q.Q., Zhang K.K., Zhu Q.Y., Li Z.J., Liu Y.J., Fitzek E., Yohe T., Zhao L.M., Li W.H., Liu T. (2018). Structural and biochemical characterization of a multidomain alginate lyase reveals a novel role of CBM32 in CAZymes. Biochim. Biophys. Acta-Gen. Subj..

[14] Jouanneau D., Klau L.J., Larocque R., Jaffrennou A., Duval G., Le Duff N., Roret T., Jeudy A., Aachmann F.L., Czjzek M. (2021). Structure-function analysis of a new PL17 oligoalginate lyase from the marine bacterium *Zobellia galactanivorans* Dsij^T^. Glycobiology.

[15] Kikuchi M., Konno N., Suzuki T., Fujii Y., Kodama Y., Isogai A., Habu N. (2020). A bacterial endo-β-1,4-glucuronan lyase, CUL-I from *Brevundimonas* sp. SH203, belonging to a novel polysaccharide lyase family. Protein Expr. Purif..

[16] Pilgaard B., Vuillemin M., Munk L., Holck J., Meier S., Wilkens C., Meyer A.S. (2022). Discovery of a novel glucuronan lyase system in *Trichoderma parareesei*. Appl. Environ. Microbiol..

[17] Li M.M., Shang Q.S., Li G.S., Wang X., Yu G.L. (2017). Degradation of marine algae-derived carbohydrates by *Bacteroidetes* isolated from human gut microbiota. Mar. Drugs.

[18] Tang J.C., Taniguchi H., Chu H., Zhou Q., Nagata S. (2009). Isolation and characterization of alginate-degrading bacteria for disposal of seaweed wastes. Lett. Appl. Microbiol..

[19] Wang M.P., Chen L., Liu Z.Y., Zhang Z.J., Qin S., Yan P.S. (2016). Isolation of a novel alginate lyase-producing *Bacillus litoralis* strain and its potential to ferment *Sargassum horneri* for biofertilizer. Microbiologyopen.

[20] An Q.D., Zhang G.L., Wu H.T., Zhang Z.C., Zheng G.S., Luan L., Murata Y., Li X. (2009). Alginate-deriving oligosaccharide production by alginase from newly isolated *Flavobacterium* sp. LXA and its potential application in protection against pathogens. J. Appl. Microbiol..

[21] Ito M., Watanabe K., Maruyama T., Mori T., Niwa K., Chow S., Takeyama H. (2019). Enrichment of bacteria and alginate lyase genes potentially involved in brown alga degradation in the gut of marine gastropods. Sci. Rep..

[22] Erasmus J.H., Cook P.A., Coyne V.E. (1997). The role of bacteria in the digestion of seaweed by the abalone *Haliotis midae*. Aquaculture.

[23] Bansemer M.S., Qin J.G., Harris J.O., Howarth G.S., Stone D.A.J. (2016). Nutritional requirements and use of macroalgae as ingredients in abalone feed. Rev. Aquac..

[24] Tanaka R., Shibata T., Miyake H., Mori T., Tamaru Y., Ueda M., Bossier P. (2016). Temporal fluctuation in the abundance of alginate-degrading bacteria in the gut of abalone *Haliotis gigantea* over 1 year. Aquac. Res..

[25] Kurahashi M., Yokota A. (2004). *Agarivorans albus* gen. nov., sp. nov., a gamma-proteobacterium isolated from marine animals. Int. J. Syst. Evol. Microbiol..

[26] Long M.X., Yu Z.N., Xu X. (2010). A novel beta-agarase with high ph stability from marine *Agarivorans* sp. LQ48. Mar. Biotechnol..

[27] Du Z.J., Wang J., Yang L.J., Chen G.J. (2011). Identification of a marine agarolytic bacterium *Agarivorans albus* QM38 and cloning and sequencing its beta-agarase genes. Acta Oceanol. Sin..

[28] Lee D.G., Jeon M.J., Lee S.H. (2012). Cloning, expression, and characterization of a glycoside hydrolase family 118 beta-agarase from *Agarivorans* sp. JA-1. J. Microbiol. Biotechnol..

[29] Miller G.L. (1959). Use of dinitrosalicylic acid reagent for determination of reducing sugar. Anal. Chem..

[30] Li S.Y., Yang X.M., Bao M.M., Wu Y., Yu W.G., Han F. (2015). Family 13 carbohydrate-binding module of alginate lyase from *Agarivorans* sp. L11 enhances its catalytic efficiency and thermostability, and alters its substrate preference and product distribution. Fems Microbiol. Lett..

[31] Li S.Y., Yang X.M., Zhang L., Yu W.G., Han F. (2015). Cloning, expression, and characterization of a cold-adapted and surfactant-stable alginate lyase from marine bacterium *Agarivorans* sp. L11. J. Microbiol. Biotechnol..

[32] Uchimura K., Miyazaki M., Nogi Y., Kobayashi T., Horikoshi K. (2010). Cloning and sequencing of alginate lyase genes from deep-sea strains of *Vibrio* and *Agarivorans* and characterization of a new *Vibrio* enzyme. Mar. Biotechnol..

[33] Kobayashi T., Uchimura K., Miyazaki M., Nogi Y., Horikoshi K. (2009). A new high-alkaline alginate lyase from a deep-sea bacterium *Agarivorans* sp.. Extremophiles.

[34] Hisano T., Nishimura M., Yamashita T., Imanaka T., Muramatsu T., Kimura A., Murata K. (1994). A simple method for determination of substrate-specificity of alginate lyases. J. Ferment. Bioeng..

[35] Al-Saari N., Gao F., Rohul A., Sato K., Sato K., Mino S., Suda W., Oshima K., Hattori M., Ohkuma M. (2015). Advanced microbial taxonomy combined with genome-based-approaches reveals that *Vibrio astriarenae* sp. nov., an agarolytic marine bacterium, forms a new clade in *Vibrionaceae*. PLoS ONE.

[36] Liu Y.P., Jin X.K., Wu C., Zhu X.Y., Liu M., Call D.R., Zhao Z. (2020). Genome-wide identification and functional characterization of β-agarases in *Vibrio astriarenae* strain HN897. Front. Microbiol..

[37] El Rayes J., Rodriguez-Alonso R., Collet J.F. (2021). Lipoproteins in Gram-negative bacteria: New insights into their biogenesis, subcellular targeting and functional roles. Curr. Opin. Microbiol..

[38] Srisodsuk M., Reinikainen T., Penttila M., Teeri T.T. (1993). Role of the interdomain linker peptide of trichoderma-reesei cellobiohydrolase-i in its interaction with crystalline cellulose. J. Biol. Chem..

[39] Wang Z., Zhang T.R., Long L.K., Ding S.J. (2018). Altering the linker in processive GH5 endoglucanase 1 modulates lignin binding and catalytic properties. Biotechnol. Biofuels.

[40] Dubois M., Gilles K.A., Hamilton J.K., Rebers P.A., Smith F. (1956). Colorimetric method for determination of sugars and related substances. Anal. Chem..

[41] Yoon S.H., Ha S.M., Lim J., Kwon S., Chun J. (2017). A large-scale evaluation of algorithms to calculate average nucleotide identity. Antonie Van Leeuwenhoek.

[42] Meier-Kolthoff J.P., Carbasse J.S., Peinado-Olarte R.L., Goker M. (2022). TYGS and LPSN: A database tandem for fast and reliable genome-based classification and nomenclature of prokaryotes. Nucleic Acids Res..

[43] Smibert R., Krieg N., Gerhardt P., Murray R.G.E., Wood W.A., Krieg N.R. (1994). Phenotypic characterization. Methods for General and Molecular Bacteriology.

[44] Bowman J.P. (2000). Description of *Cellulophaga algicola* sp. nov., isolated from the surfaces of Antarctic algae, and reclassification of *Cytophaga uliginosa* (ZoBell and Upham 1944) Reichenbach 1989 as *Cellulophaga uliginosa* comb. nov. Int. J. Syst. Evol. Microbiol..

[45] Dong X.-Z., Cai M.-Y. (2001). Determinative Manual for Routine Bacteriology.

[46] Edgar R.C., Soc I.C. MUSCLE: Multiple sequence alignment with improved accuracy and speed. Proceedings of the IEEE Computational Systems Bioinformatics Conference (CSB 2004).

[47] Capella-Gutierrez S., Silla-Martinez J.M., Gabaldon T. (2009). trimAl: A tool for automated alignment trimming in large-scale phylogenetic analyses. Bioinformatics.

[48] Trifinopoulos J., Nguyen L.T., von Haeseler A., Minh B.Q. (2016). W-IQ-TREE: A fast online phylogenetic tool for maximum likelihood analysis. Nucleic Acids Res..

[49] Letunic I., Bork P. (2011). Interactive Tree Of Life v2: Online annotation and display of phylogenetic trees made easy. Nucleic Acids Res..

[50] Yoon S.H., Ha S.M., Kwon S., Lim J., Kim Y., Seo H., Chun J. (2017). Introducing EzBioCloud: A taxonomically united database of 16S rRNA gene sequences and whole-genome assemblies. Int. J. Syst. Evol. Microbiol..

[51] Thompson J.D., Gibson T.J., Plewniak F., Jeanmougin F., Higgins D.G. (1997). The CLUSTAL_X windows interface: Flexible strategies for multiple sequence alignment aided by quality analysis tools. Nucleic Acids Res..

[52] Saitou N., Nei M. (1987). The neighbor-joining method: A new method for reconstructing phylogenetic trees. Mol. Biol. Evol..

[53] Fitch W.M. (1971). Toward defining the course of evolution-minimum change for a specific tree topology. Syst. Zool..

[54] Felsenstein J. (1981). Evolutionary trees from DNA sequences: A maximum likelihood approach. J. Mol. Evol..

[55] Kumar S., Stecher G., Tamura K. (2016). MEGA7: Molecular evolutionary genetics analysis version 7.0 for bigger datasets. Mol. Biol. Evol..

[56] Kimura M. (1980). A simple method for estimating evolutionary rates of base substitutions through comparative studies of nucleotide-sequences. J. Mol. Evol..

[57] Zhang H., Yohe T., Huang L., Entwistle S., Wu P.Z., Yang Z.L., Busk P.K., Xu Y., Yin Y.B. (2018). dbCAN2: A meta server for automated carbohydrate-active enzyme annotation. Nucleic Acids Res..

[58] Yin Y.B., Mao X.Z., Yang J.C., Chen X., Mao F.L., Xu Y. (2012). dbCAN: A web resource for automated carbohydrate-active enzyme annotation. Nucleic Acids Res..

[59] Yang J.Y., Yan R.X., Roy A., Xu D., Poisson J., Zhang Y. (2015). The I-TASSER Suite: Protein structure and function prediction. Nat. Methods.

[60] Zhang Y., Skolnick J. (2005). TM-align: A protein structure alignment algorithm based on the TM-score. Nucleic Acids Res..

[61] Juncker A.S., Willenbrock H., Von Heijne G., Brunak S., Nielsen H., Krogh A. (2003). Prediction of lipoprotein signal peptides in Gram-negative bacteria. Protein Sci..

[62] Savojardo C., Martelli P.L., Fariselli P., Profiti G., Casadio R. (2018). BUSCA: An integrative web server to predict subcellular localization of proteins. Nucleic Acids Res..

