# Supplementary material for: Degradation of Alginate by a Newly Isolated Marine Bacterium Agarivorans sp. B2Z047
Source: Mar Drugs. 2022 Apr 4;20(4):254. doi: 10.3390/md20040254 (PMC9029943; doi:10.3390/md20040254)
Supplement: Supplementary file 1 [file marinedrugs-20-00254-s001.zip › Supplementary Materials.pdf]

Supplementary materials

Degradation of alginate by a newly isolated marine bacterium *Agarivorans* sp. B2Z047

Xun-Ke Sun[1], Ya Gong[1,2,*], Dan-Dan Shang[1], Bang-Tao Liu[1], Zong-Jun Du[1,2] and Guan-Jun Chen[1,2,*]

1 Marine College, Shandong University, Weihai 264209, China.
2 State key Laboratory of Microbial Technology, Shandong University, Qingdao 266237, China.
* Correspondence: gongya@sdu.edu.cn, guanjun@sdu.edu.cn

Content

Data S1

Figure S1 and S2

Table S1, S2, S3 and S4

Data S1. Information of 100 alginate-degrading strains.

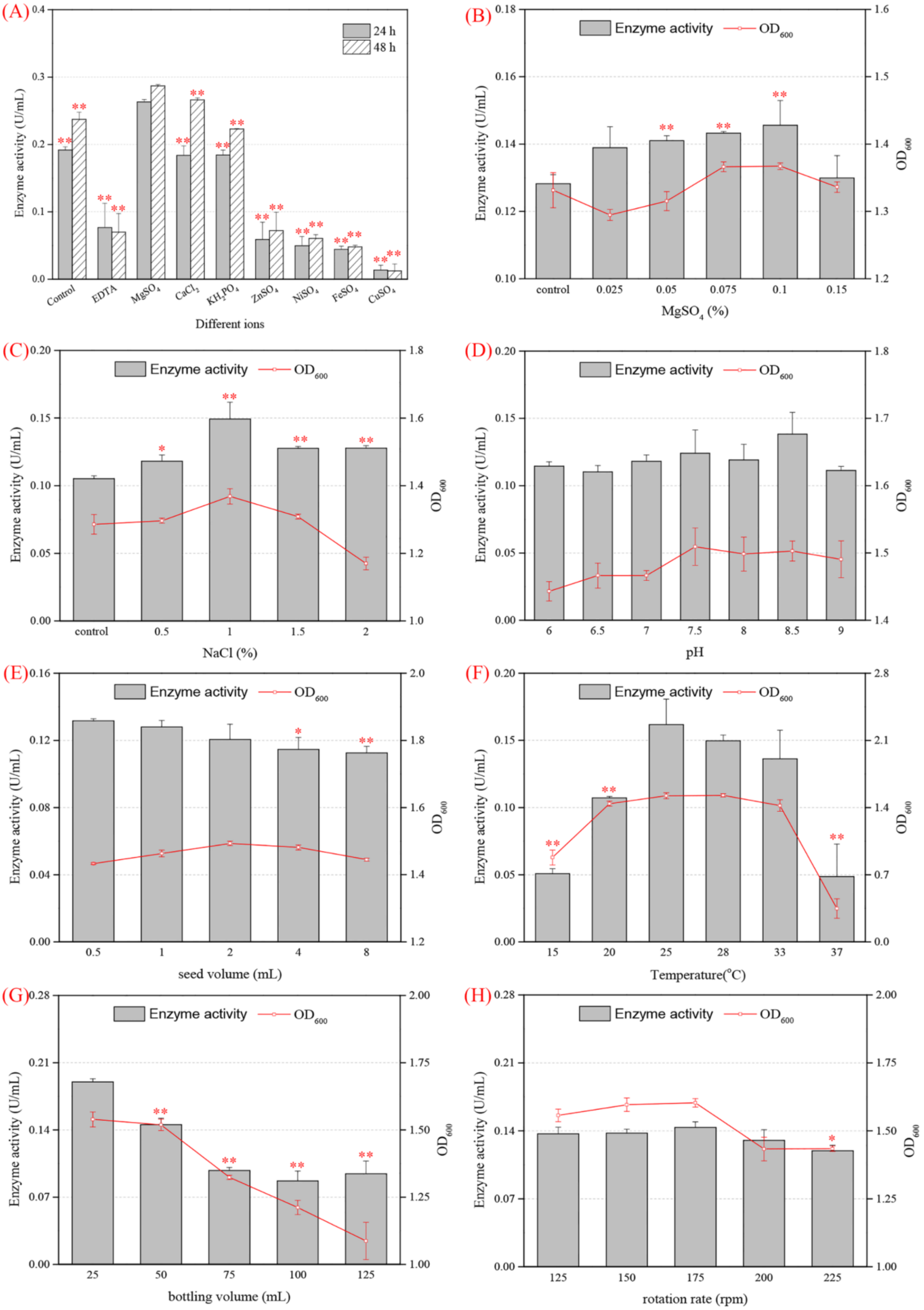

Figure S1. Optimization of alginate lyase production conditions of strain B2Z047. Influences of different ions (**A**), different concentrations of $MgSO_4$ (**B**) and NaCl (**C**), initial pHs of the culture media (**D**), seed

volumes (**E**), culture temperatures (**F**), bottling volumes in the flasks (**G**), and rotation speed of shaker (**H**) on the alginate lyase production. The optimum culture conditions were established on the optimal medium and all experiments were performed with three biological replicates each time and repeated more than three times. Alginate lyase activity and biomass were measured after 24 h cultivation. All medium were prepared with artificial seawater (3% NaCl, 0.23% $MgCl_2$, 0.32% $MgSO_4$, 0.12% $CaCl_2$, 0.07% KCl, and 0.02% $NaHCO_3$, w/v). The concentration of each salt added was 0.1% (**A**). The NaCl was supplemented, for example, 1% NaCl addition producing final NaCl concentration of 4% (**C**). The error bars represented the standard deviation, and asterisks denoted *p*-values for t-tests of differences from the data of $MgSO_4$ (**A**), control (**B & C**), 0.5 mL (**E**), 28°C (**F**), 25 mL (**G**) and 175 rpm (**H**): $^{*}p < 0.05$, $^{**}p < 0.01$.

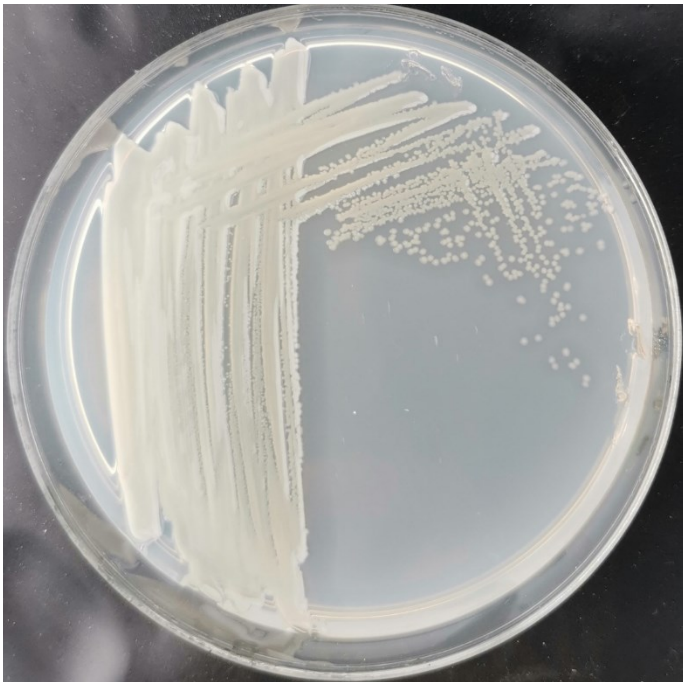

Figure S2. The colony morphology of strain B2Z047 grew on MA plate at 30°C for two days.

Table S1. Four factors and three levels of orthogonal experiment.

Level	Factor			
	Sodium alginate (A, %)	Yeast extract (B, %)	NaCl (C, %)	$MgSO_4$ (D, %)
1	0.7	0.3	0	0.05
2	0.9	0.5	1	0.1
3	1.1	0.7	2	0.2

The results and analyses of orthogonal experiment:

NO.	A	B	C	D	Enzyme activity
1	1	1	1	1	0.1307
2	2	2	2	1	0.1319
3	3	3	3	1	0.1226
4	3	2	1	2	0.1333
5	2	1	3	2	0.1473
6	1	3	2	2	0.1264
7	1	2	3	3	0.1221
8	2	3	1	3	0.1085
9	3	1	2	3	0.1596
K1	0.3792	0.4376	0.3725	0.3852	
K2	0.3877	0.3873	0.4179	0.407	
K3	0.4155	0.3575	0.3920	0.3902	
R	0.0363	0.08	0.0454	0.0168	
Factor order	B>C>A>D				
Excellent level	A3	B1	C2	D2	
Excellent combination	A3B1C2D2				

Ki (1,2,3) is obtained by adding any number of columns corresponding to i factor.

R is the difference between the maximum value and the minimum value of Ki (1,2,3) of any columns.

Table S2. Function of the closest structure and structural similarity (TM-score) of alginate lyases.

Protein Name	PDB code	TM-score	Function
**Aly7A1**	4ozx	0.859	alginate lyase from *Klebsiella pneumoniae*
Aly7A2		0.813	
Aly7A3		0.785	
Aly7A4		0.856	
Aly7A5		0.972	
**Aly7B1**	5zu5	0.979	a full length alginate lyase with CBM domain from *Vibrio splendidus*
Aly7B2		0.913	
**Aly7C**		0.853	
**Aly6A**	7dmk	0.827	PL6 alginate lyase from *Bacteroides clarus*
**Aly38A**	3nfv	0.897	alginate lyase from *Bacteroides ovatus*
**Oal17A1**	7bjt	0.981	a new PL17 oligoalginate lyase from *Zobellia galactanivorans*
Oal17A2		0.990	

The model structures highlighted in bold type are showed in Figure 4.

Table S3. Genes for the alginate-degrading process in the genome of *Agarivorans* sp. B2Z047.

Gene locus	E-Value	Accession	Short name	Superfamily
		**outermembrane porin (KdgMN)**		
2224	4.76606E-35	pfam06178	KdgM oligogalacturonate-specific porin	cl11629
2251	2.34382E-34	pfam13778	DUF4174	cl16390
		**oligoalginate transporter (ToaABC)**		
1830	0	TIGR02121	Na_Pro_sym	cl00456
2233	2.2246E-107	cd11477	SLC5sbd_u1	cl00456
2919	6.92446E-110	cd11477	SLC5sbd_u1	cl00456
4486	3.64829E-66	COG0591	PutP	cl00456
		**DEH reductase (DehR)**		
345	1.99E-73	COG2084	MmsB	cl34402
1772	2.2E-104	COG2084	MmsB	cl34402
		**ED PATHWAY: KDG kinase**		
1151	5.27E-94	cd01166	KdgK	cl00192
2200	0	PRK15074	PRK15074	cl00192
2235	1.42E-89	cd01166	KdgK	cl00192
3592	7.22E-15	cd01166	KdgK	cl00192
4161	1.9E-47	cd01166	KdgK	cl00192
		**ED PATHWAY: KDPGQ aldolase**		
1150	1.32E-75	cd00452	KDPG_aldolase	cl21457
2236	1.5E-81	cd00452	KDPG_aldolase	cl21457

Table S4. Primers used in this study.

Primers	Sequences（5′–3′）
**Primers for the gene expression in *E. coli***	
Aly6A-28a-F	GGAATTCTGTAATAGCAGTAGCGAC
Aly6A-28a-R	ATAAGAATGCGGCCGCGTCTTCGCGACTCATTAATC
Aly7A1/7A4-28a-F	GGAATTCTTCTCCGGGGATTTAAAA
Aly7A1/7A4-28a-R	ATAAGAATGCGGCCGCTCGGTGGAAAAGGTTGATT
Aly7A2-28a-F	GGAATTCGCGAATACACCGACCAATTA
Aly7A2-28a-R	ATAAGAATGCGGCCGCTTGAATAAGGTTAAGTTTA
Aly7A3-28a-F	GGAATTCAATGTTCAGTTTTCTGATAAC
Aly7A3-28a-R	ATAAGAATGCGGCCGCTTTAGGCTCAGCTGCTGAGTACT
AlyA5-28a-F	GGAATTCGCCACGACTACGCCAGCAGAAG
AlyA5-28a-R	ATAAGAATGCGGCCGCCTTAGCAGAGCCGTGGTTTACAT
Aly7B1-28a-F	GGAATTCTGTACATCAACTTCTACTCCT
Aly7B1-28a-R	ATAAGAATGCGGCCGCTTTCTCAGTGTAACTATCGTG
Aly7B2-28a-F	GGAATTCTGTGGCGGTTCTTCTGAAAC
Aly7B2-28a-R	ATAAGAATGCGGCCGCAGGATTATAACCTTGGTG
Aly7C-28a-F	GGAATTCGCAACTTTCAACATGCAAAAGA
Aly7C-28a-R	ATAAGAATGCGGCCGCTGGGTTGTAGTTATCGTGAG
Oal17A1-28a-F	GGAATTCACTTACCAACCATTATTGA
Oal17A1-28a-R	ATAAGAATGCGGCCGCAGCTATGTGAACCGCGCCACTCC
Oal17A2-28a-F	GGAATTCCTTGCGAATTTAAATGCA
Oal17A2-28a-R	ATAAGAATGCGGCCGCACCTCGGTTCTTTTCTAAC
Aly38A-28a-F	GGAATTCGCCGAATTTGTATTTGTGAAAG
Aly38A-28a-R	ATAAGAATGCGGCCGCTTGTTTTCGGTATAACAGTTCT
**Primers for q-PCR**	
16S-F	CCTTACCTGCCCTTGACATA
16S-R	CGGGACTTAACCCAACATC
Aly6A-F	ATGTATGCGGGAAGTAGCGACAAC
Aly6A-R	GAGATAACCTCGGCTTCACCTTGG
Aly7A1/7A4-F	GATTTGGGTGGCGGCGTGAC
Aly7A1/7A4-R	TGTTGAGCAAGGGTTACTCGTGTC
AlyA2-F	CAGCAGCTTACGGCTCTATT
AlyA2-R	CACAGAGTGAGCACCCATTT

AlyA3-F	GCCGGTTAACTATCCTCAGTTT
AlyA3-R	TCTCCATCTAGCGCAAAGTATTC
AlyA5-F	CGCACTAAGCACCAGCCTATCTG
AlyA5-R	GGGCGGTCACCATTGTCCAAAG
Aly7B1-F	CGGCGGTATTGATGGCGTTCTAG
Aly7B1-R	GACCTACTTCGTTAGCAGCACCAG
Aly7B2-F	GTGGCAGGATGTAGTGAC
Aly7B2-R	ATGGTGGTAAAGATGGGT
Aly7C-F	CGAACCTATCCGCCTTTACTAC
Aly7C-R	TCGTTACCACCGTCTGATTTC
Oal17A1-F	GACCAAGCCAGCGTAGATAAA
Oal17A1-R	CCTTCCATGTAGTAACCGTCAG
Oal17A2-F	GTGGCAGCGGTAGGTATTT
Oal17A2-R	CTAAGAACCCACCCGTAACATC
Aly38A-F	AAGGACGACGCGGAGGAATTATTG
Aly38A-R	CAAGCGGGAGATTGAGACACCATC

* Restriction sites are underlined.
